# LONGITUDINAL ASSOCIATION OF SERUM CREATININE- AND CYSTATIN-C–BASED INDICES WITH INCIDENT SARCOPENIA

**DOI:** 10.1093/geroni/igad104.3456

**Published:** 2023-12-21

**Authors:** Jae Young Jang, Hyung Eun Shin, Chang Won Won, Miji Kim

**Affiliations:** Kyung Hee University, Seoul, Republic of Korea; Kyung Hee University, Seoul, Republic of Korea; Kyung Hee University Hospital, Seoul, Republic of Korea; Kyung Hee University Hospital, Seoul, Republic of Korea

## Abstract

Although a cross-sectional link between serum creatinine- and cystatin-C-based indices and sarcopenia has been established, the longitudinal association remains unclear. This study explored the longitudinal association between serum creatinine- and cystatin-C-based indices and the incidence of sarcopenia in community-dwelling older adults. Using a 2–4 year follow-up data from the Korean Frailty and Aging Cohort Study, 690 participants (mean age: 75.2±3.7 years; 45.9% men) without sarcopenia at baseline were analyzed. The association between the incidence of sarcopenia during a 4-year follow-up and serum creatinine-to-cystatin-C ratio, estimated glomerular filtration rate (eGFR) ratio (eGFRcystatin-C/eGFRcreatinine), sarcopenia index (serum creatinine×eGFRcystatin-C), predicted skeletal muscle mass index (pSMI), and total-body muscle mass index (TBMM) from baseline were compared. During the 4-year follow-up period, the incidence of sarcopenia was 21.5% in men and 14.7% in women. The pSMI and TBMM showed a higher area under the receiver operating characteristics curves (AUC) for predicting the incidence of sarcopenia in men (pSMI, AUC:0.616, p=0.003; TBMM, AUC:0.613, p=0.001) and women (pSMI, AUC:0.714, p=0.003; TBMM, AUC:0.714, p=0.001) than other indices. After adjusting potential confounders, higher pSMI and TBMM were associated with sarcopenia onset in men (pSMI, odds ratio [OR]: 0.417, 95% confidence interval [CI]: 0.237–0.733; TBMM, OR: 0.937, 95%CI: 0.901–0.975) and women (pSMI, OR: 0.166, 95%CI: 0.072–0.380; TBMM, OR: 0.906, 95%CI: 0.862–0.952). In conclusion, pSMI and TBMM demonstrated accurate predictive capabilities for sarcopenia. Higher pSMI and TBMM values were associated with sarcopenia incidence. These findings suggest pSMI and TBMM could be potential predictive biomarkers of sarcopenia in community-dwelling older adults.

